# Nebulized pharmacological agents for preventing postoperative sore throat: A systematic review and network meta-analysis

**DOI:** 10.1371/journal.pone.0237174

**Published:** 2020-08-10

**Authors:** Jian Yu, Li Ren, Su Min, You Yang, Feng Lv

**Affiliations:** Department of Anesthesiology, the First Affiliated Hospital of Chongqing Medical University, Chongqing, China; AUSL della Romagna, ITALY

## Abstract

Postoperative sore throat is one of the most common complications following endotracheal intubation. Nebulization therapy, a preferable and safety method of drug delivery, has been shown to be effective in postoperative sore throat prevention in many studies. However, the relative efficacy of various nebulized agents remains unknown. In this review, we aimed to quantify and rank order the efficacy of available nebulized agents for various postoperative sore throat-related outcomes. A comprehensive literature search of PubMed, EMBASE, CENTRAL and Google Scholar was conducted to identify eligible studies from inception to 25 May 2020. Incidence of postoperative sore throat 1hour and 24hours postoperatively and severity of postoperative sore throat 24 hours postoperatively were the primary outcomes. We conducted a Bayesian network meta-analysis to combine direct and indirect evidence to estimate the relative effects between treatments as well as the probabilities of ranking for treatments based on their protective effects. We identified 32 trials assessing 6 interventions. Overall inconsistency and heterogeneity were acceptable. Nebulized corticosteroids, magnesium, and ketamine differed from non-analgesic methods on the three primary outcomes. Based on the surface under the cumulative ranking curve, nebulized corticosteroids ranked first in almost all outcomes among the nebulized drugs. Considering only high-quality and 2-arm design studies, nebulized corticosteroids still seemed best. In conclusion, prophylactic use of nebulized corticosteroids, magnesium, and ketamine can effectively prevent postoperative sore throat, and nebulized corticosteroids appears to be the overall best approach.

## Introduction

Endotracheal intubation is one of the most common procedures under general anesthesia (GA), but it can lead to a series of airway complications. Postoperative sore throat (POST) is one of the most undesirable outcomes that occurs in approximately 62% or more patients following GA[1–6]. Mechanical injury during intubation, damage to the mucosa due to the pressure from the endotracheal tube(ETT) cuff and dehydration of the mucosa were considered as the cause of POST[3]. Although it is self-limiting, it needs to be prevented because it seriously affects patient satisfaction and postoperative recovery[7].

Nebulization therapy is widely used to prevent POST. It is highly recommended because of a small volume of drugs required for effect, easy way of administration, better patient compliance, and most importantly little risk of adverse events compared with other methods(such as gargle, intravenous, etc.)[8]. Commonly used aerosolized drugs include corticosteroids, ketamine, magnesium, lidocaine, Non-steroidal anti-inflammatory drugs(NSAIDs), etc., and have achieved various effects[9–12]. Despite the continuous emergence of clinical trials on POST nebulization therapy, there is still no current overview of all the relevant drugs. There is a lack of direct comparisons of frequently used aerosolized drugs and a lack of a clinically useful ranking of all atomizing drugs with respect to both efficacy and acceptability.

Therefore, we conducted a systematic review and network meta-analysis (NMA) to allows comparison of different nebulized agents in a direct and indirect manner. The aim of this review is to generate a clinically useful ranking of nebulized drugs to guide clinical decisions. We focused on the incidence and severity of POST, and reported adverse events, postoperative cough and hoarseness.

## Materials and methods

This review was developed following the preferred reporting items for systematic review and meta-analyses (PRISMA) extension statement for NMA[13](S1 Table). The protocol for this systematic review and NMA has been registered at PROSPERO(CRD42020171703)(S1 Appendix).

### Search strategy

We performed a systematic search of PubMed, EMBASE, and the Cochrane Central Register of Controlled Trials(CENTRAL) for eligible studies. We reviewed the reference lists of the included publications and previous systematic reviews, and searched Google Scholar to identify further eligible studies. No language, year of publication or publication status restrictions were imposed. The search strategy is shown in S2 Appendix. The search was updated on May 25, 2020.

### Eligibility criteria

Two reviewers (JY and YY) independently evaluated the studies for eligibility. Disagreements between the reviewers concerning the decision to include or exclude a study were resolved by consensus, and if necessary, consultation with a third reviewer (SM). Studies were selected when they were as follows: (1)Studies with randomized controlled study design; (2)Patients who received general anesthesia and endotracheal intubation; (3)Nebulized drugs usage before anesthesia. Exclusion criteria were as follows: (1)Studies not reporting airway complications; (2)Patients underwent head and neck surgery or used laryngeal mask airway; (3)After team discussions, we believe that drugs sprayed on the distal end or the cuff of the ETT are more similar to lubrication than to nebulization, so we excluded such studies.

### Data extraction

Two investigators (JY and YY) independently extracted data from original texts and supplementary appendix using a data abstraction form. The following information such as first author, year of publication, country of origin, sample size, age, gender, ASA, size of ETT, cuff pressure, endotracheal intubation technique, time of operation or anesthesia, type of surgery, interventions used, and relevant outcomes were collected. The primary outcomes were the incidence of POST 1 hour and 24 hours after surgery/extubation, and the incidence of moderate to severe POST 24 hours after surgery/extubation, if information at 1 hour was not available, we used data ranging between 0 and 2 hours(if equidistant, we took the longer outcome). Because there is currently no uniformly definition for moderate to severe POST, we have adopted the commonly used 4-point scale(0–3) for evaluation and we elected to choose a threshold of at least two as the definition for moderate to severe POST[14]. The secondary outcomes were the incidence of postoperative cough and postoperative hoarseness 24h after surgery/extubation, and adverse effects. When there was no data available in the study or no original data was acquired from the authors, the data was obtained from other/published articles. Disagreements between the two reviewers were resolved by consensus, and if necessary, by consultation with a third reviewer (SM).

### Quality assessment

Two reviewers (JY and YY) independently performed the quality assessment. We used the Cochrane risk of bias assessment tool to assess the risk of bias (including selection, performance, detection, attrition and reporting bias among the included randomized trials)[15]. Blinding remains critical for the quality of study, especially for those with high subjective outcomes, such as pain or depression assessment[16]. Thus, the studies with high or unclear risk of performance bias (blinding of participants) were classified as low quality. We also checked for industry sponsorship or conflicts of interest. Risk of bias tables were created in RevMan5.3[17].

### Statistical analysis

The evaluation of airway complications outcomes was based on the synthesis of data extracted from included trials, then combine direct and indirect comparisons to estimate the overall effects among multiple nebulized agents. In this network meta-analysis we used the random-effects model and conducted in Bayesian framework. The effects of nebulized agents on airway complications outcomes were analyzed using the odds ratios (OR) and 95% credible interval (CI). The OR > 1.0 were indicated as higher risk, the CI which did not include 1.0 was considered to be statistically significant. All the analyses were generated by R Software with GeMTC package[18, 19]. The inconsistency of network meta-analysis was assessed using the node-splitting models to detect whether the results of direct and indirect comparison were in agreement within treatment loops[20]. The node-splitting models cannot be performed when the outcome which lacked direct or indirect comparison. Thus, we used the analysis of heterogeneity to quantify the degree of heterogeneity by I2 calculation. The values of I2 > 50% was considered heterogeneity across the trials. Ranking followed probability score (P score) calculation[21, 22] from the surface under the cumulative ranking curve (SUCRA) method. Generally, SUCRA values are interpreted as probabilities, and the larger the probability, the better the treatment. For sensitivity analysis, we considered incidence of POST 24h operatively since we considered it the single most important outcome and was the one most often reported. Our sensitivity analyses consisted of excluding low quality studies, and non 2-arm design studies to explore whether study quality and design inconsistency might affect overall effect.

## Result

### Study selection and characteristics

**Fig 1:**
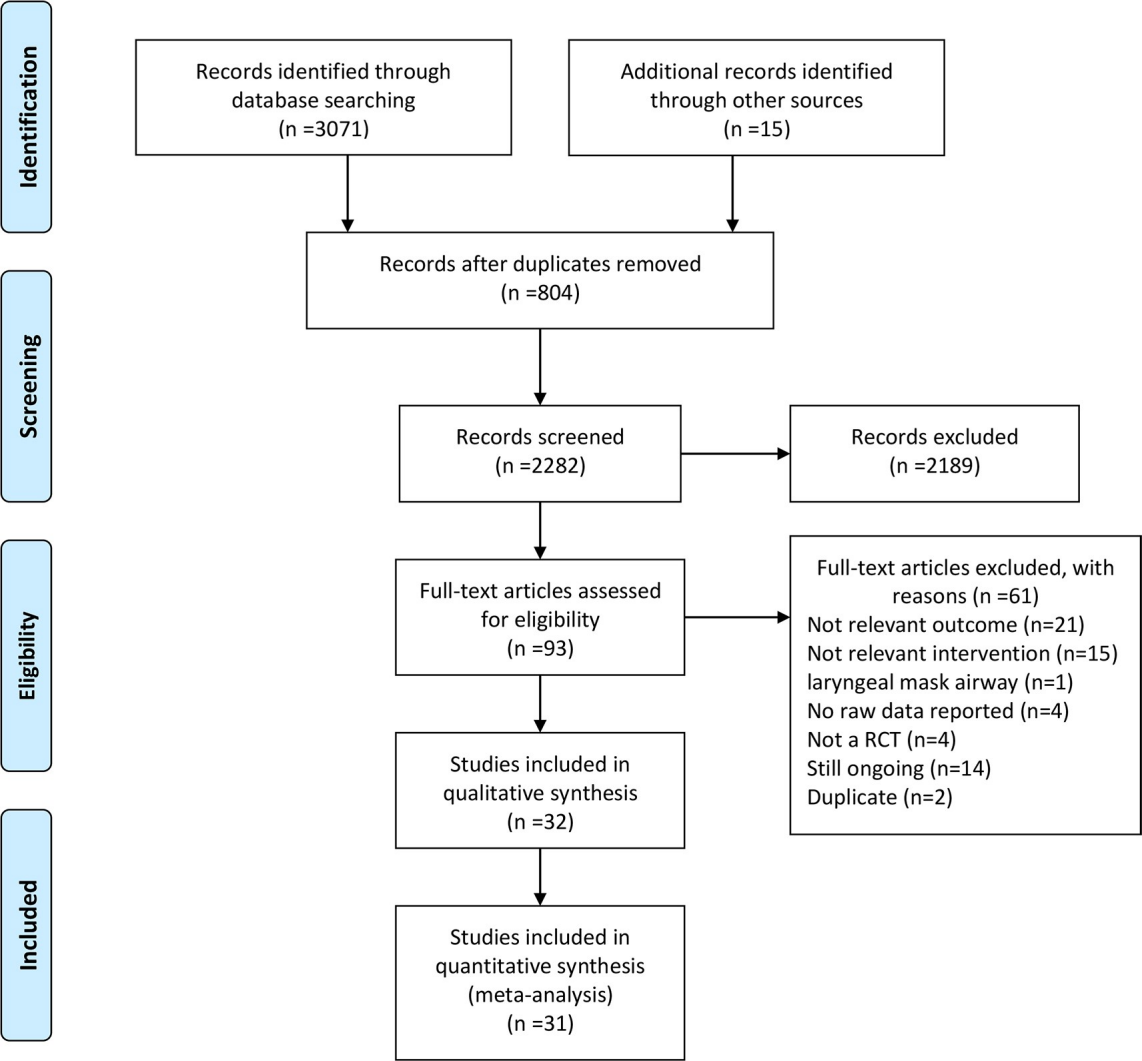
Flow diagram of the literature review.

A total of 3071 references were identified using the search strategies. and an additional search found 15 articles. After removing 804 duplicates, 2282 studies were selected through titles and abstracts screening. Full texts were obtained for further evaluation. After pre-screening, 2192 studies were excluded due to unsatisfying the inclusion criteria. Finally, a total of 32 studies fulfilled the inclusion criteria and were reviewed in the present network meta-analysis. The range of publication year was 2002–2020. The flow diagram for results of the electronic search was described in Fig 1.

The characteristics of included studies were shown in S2 Table. The mean age of the included 3732 participants was 39.7 years old and the proportion of female patients ranged from 22% to 100%. All studies were conducted in patients with endotracheal intubation, including 31 single-lumen endotracheal intubation and 1 double-lumen endotracheal intubation. Except for 8 trials that did not report intubation techniques, direct laryngoscope was used in almost all the other trials. 23 of the single-lumen endotracheal intubation studies used ETT sizes in the range of <8.5mm for men and <7.5mm for women, and only 2 studies used ETT sizes beyond that. The remaining 6 trials did not provide details on ETT sizes. 27 studies included patients with ASA physical status 1–2 and 4 included patients with ASA physical status 1–3. In most studies, drugs were administered before intubation, while in only one study it was administered before intubation and after extubation[23]. The simple summary data for each intervention group was shown in S3 Table.

**Fig 2:**
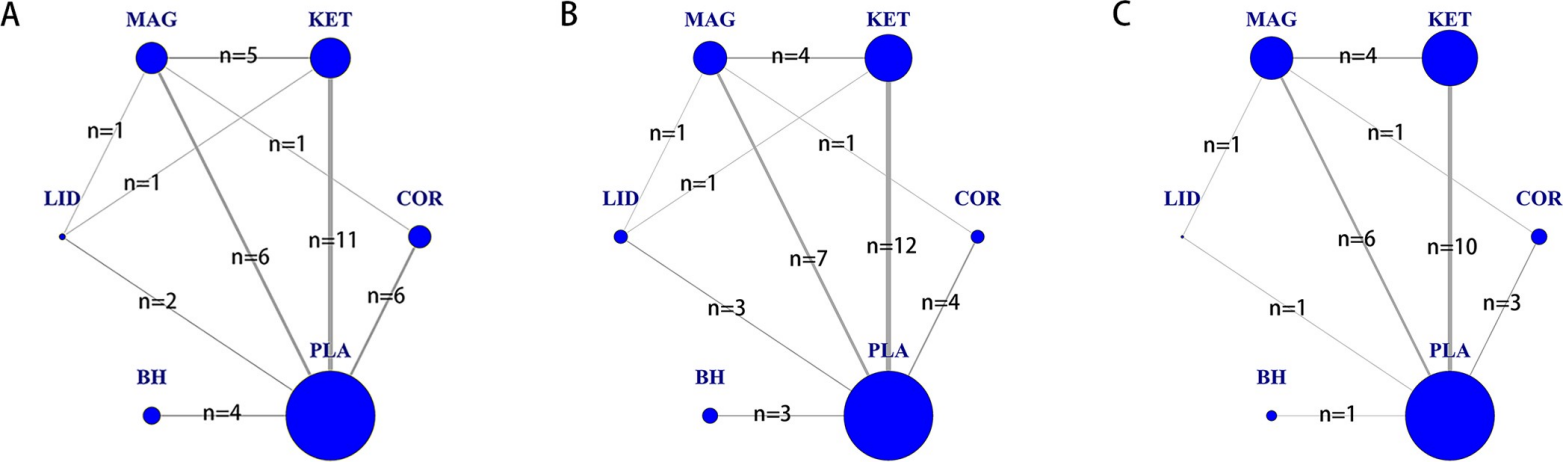
Network plot of eligible comparisons for (A) incidence of POST 24h after surgery, (B) incidence of POST 1h after surgery and (C) incidence of moderate to severe POST 24h after surgery. The size circle reflects the number of participants (sample size), and the width of the lines reflects the number of direct comparisons. n = number of trials for the direct comparisons. COR = corticosteroids; KET: ketamine; MAG: magnesium; LID: lidocaine; BH: benzydamine hydrochloride; PLA: placebo.

8(25%) trials used corticosteroids. The majority, 14(43.8%), used ketamine. 9(28.1%) trials used magnesium, 5(15.6%) trials used lidocaine and 4(12.5%) trials used benzydamine hydrochloride. Fig 2 showed the network plots of eligible comparisons for incidence of POST 24h after surgery(Fig 2A) and 1h after surgery (Fig 2B), and incidence of moderate to severe POST 24h after surgery (Fig 2C).

### Risk of bias assessment

Risk of bias within studies is presented in the S1 Fig. 23 trials(72%) included adequate sequence generation and 11 trials(34%) included adequate allocation concealment. Participants and outcome assessors were considered to be adequately blinded in 23 (72%) and 22 (69%) trials, respectively. 18 trials (56%) were deemed to be free from conflicts of interest or sponsorship bias(S2 Table).

### Synthesis of results

#### Incidence of POST 24h after extubation

**Fig 3:**
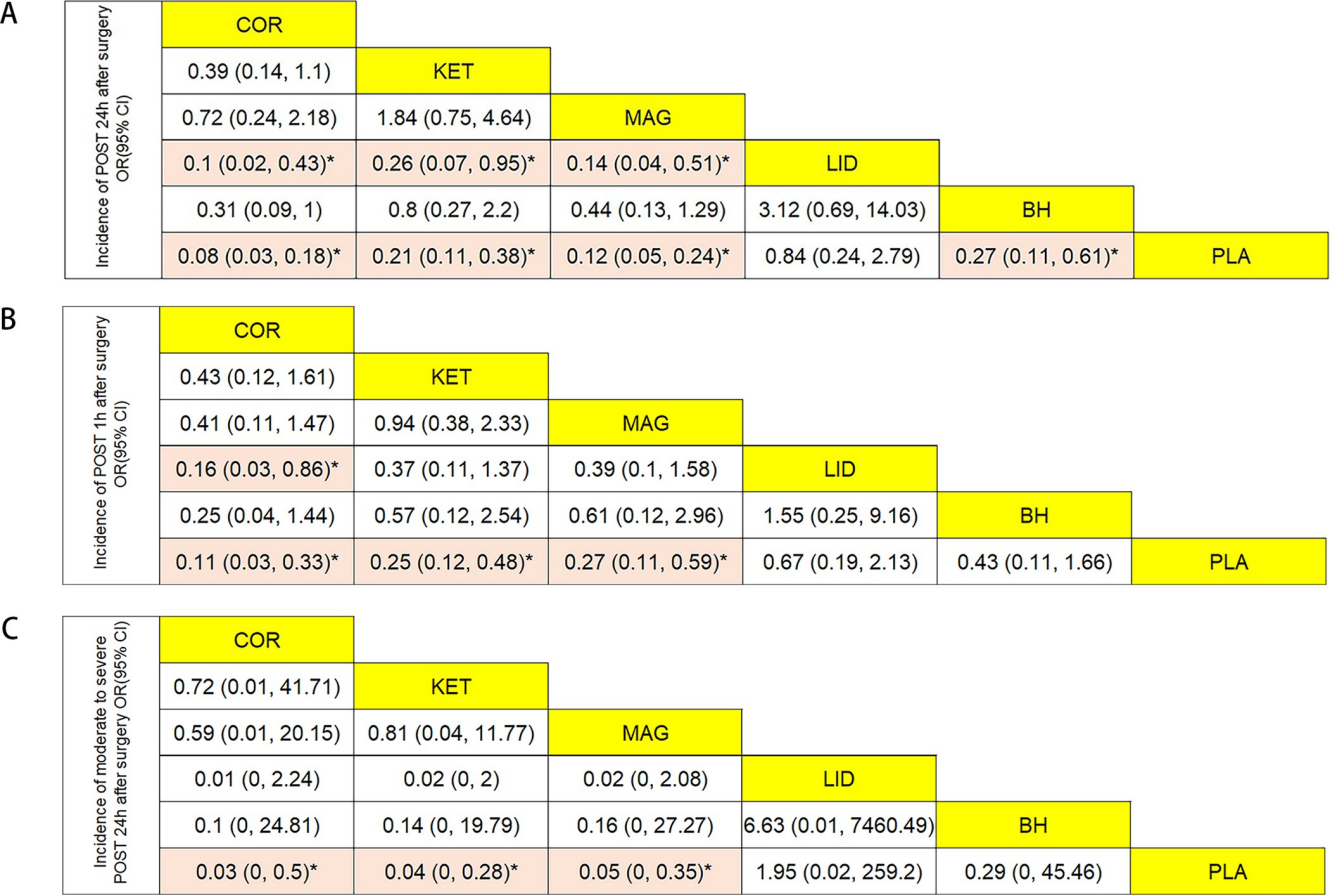
Results of the network meta-analysis for nebulized agents in terms of (A)incidence of POST 24h after surgery, (B)incidence of POST 1h after surgery and (C)incidence of moderate to severe POST 24h after surgery. Results were presented as OR with 95% CI, the estimations should read as column-defining treatment compared with the row-defining treatment. The OR below 1 was identified that the column-defining treatment had better effect on preventing POST. OR = odds ratios. CI = credible interval. * = 95% CI did not include 1. COR = corticosteroids; KET: ketamine; MAG: magnesium; LID: lidocaine; BH: benzydamine hydrochloride; PLA: placebo.

**Fig 4:**
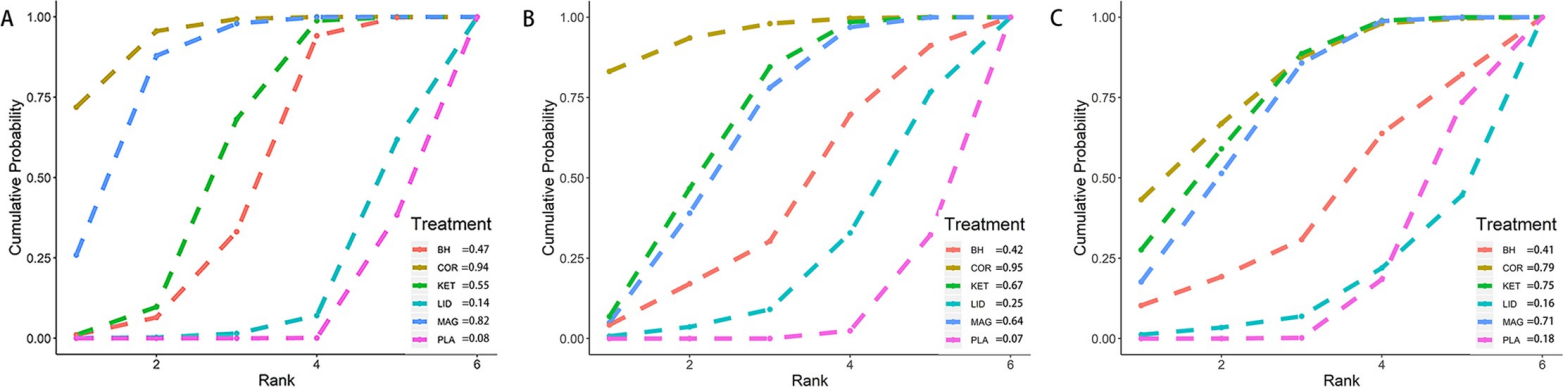
Plots of the SUCRA probabilities for nebulized agents in terms of (A)incidence of POST 24h after surgery, (B)incidence of POST 1h after surgery and (C)incidence of moderate to severe POST 24h after surgery. The area under the curve is equivalent to the value of SUCRA, and thus a bigger area corresponds to a better outcome. COR = corticosteroids; KET: ketamine; MAG: magnesium; LID: lidocaine; BH: benzydamine hydrochloride; PLA: placebo.

A total of 29 trials (2863 patients), which included 6 interventions, were included in this analysis. As shown in Fig 3A, corticosteroids(OR = 0.08, 95%CI:0.03, 0.18), ketamine(OR = 0.21, 95%CI:0.11, 0.38), magnesium(OR = 0.12, 95%CI:0.05, 0.24), and benzydamine hydrochloride(OR = 0.27, 95%CI:0.11, 0.61), these four interventions with 95%CrI excluded 1.00 had obvious advantages in reducing the incidence of POST at 24h compared with placebo. In addition, compared with lidocaine, corticosteroids(OR = 0.1, 95%CI:0.02, 0.43), ketamine(OR = 0.26, 95%CI: 0.07, 0.95), magnesium(OR = 0.14, 95%CI:0.04, 0.51) also had statistical significance. Treatment rankings based on SUCRA scores, from largest to smallest, were corticosteroids(0.94), magnesium(0.82), ketamine(0.55), benzydamine hydrochloride(0.47), lidocaine(0.14), and finally placebo (0.08)(Fig 4A). The results of node-splitting analysis showed inconsistency between the direct and indirect comparisons (S4 Table), however, the degree of heterogeneity was very low across the RCTs(I^2^ = 4%).

#### Incidence of POST 1h after extubation

A total of 28 trials (2755 patients), which included 6 interventions, were included in this analyses. As shown in Fig 3B, corticosteroids(OR = 0.11, 95%CI:0.03, 0.33), ketamine(OR = 0.25, 95%CI:0.12, 0.48), and magnesium(OR = 0.27, 95%CI:0.11, 0.59) possessed obvious strength compared with placebo. In addition, there was a statistically significant difference between corticosteroids(OR = 0.16, 95%CI:0.03, 0.86) and lidocaine. Treatment rankings based on SUCRA scores, from largest to smallest, were corticosteroids(0.95), ketamine(0.67), magnesium(0.64), benzydamine hydrochloride(0.42), lidocaine(0.25), and finally placebo (0.07) (Fig 4B). The results of node-splitting analysis did not found any inconsistency between the direct and indirect comparisons(S4 Table). There was no significant heterogeneity across the studies(I^2^ = 11%).

#### Incidence of moderate to severe POST 24h after extubation

A total of 19 trials (1963 patients), which included 6 interventions, were included in this analyses. As shown in Fig 3C, corticosteroids(OR = 0.03, 95%CI:0, 0.5), ketamine(OR = 0.04, 95%CI:0, 0.28), and magnesium(OR = 0.05, 95%CI:0, 0.35) were observed with better effects on reducing the severity of POST compared with placebo. Treatment rankings based on SUCRA scores, from largest to smallest, were corticosteroids(0.79), ketamine(0.75), magnesium(0.71), benzydamine hydrochloride(0.41), placebo (0.18), and finally lidocaine(0.16)(Fig 4C). The results of node-splitting analysis did not found any inconsistency between the direct and indirect comparisons(S4 Table). There was no significant heterogeneity across the studies(I^2^ = 0%).

#### Secondary outcomes

Postoperative cough. Only 7 trials (605 patients), which assessed 6 interventions, reported estimated postoperative cough. The rankings based on SUCRA were lidocaine(0.98), corticosteroids(0.69), magnesium(0.55), ketamine(0.33), placebo(0.22) and benzydamine hydrochloride(0.22).

*Postoperative hoarseness*. Only 8 trials (673 patients), which assessed 6 interventions, reported estimated postoperative hoarseness. The rankings based on SUCRA were corticosteroids(0.81), magnesium(0.67), benzydamine hydrochloride(0.44), lidocaine(0.42), ketamine(0.35), and placebo(0.31).

*Adverse events*. Only 9 of 32 studies included information on intervention-related adverse events. Five studies reported that none of nebulized magnesium, lidocaine and ketamine could cause adverse hemodynamic events[12, 24–27]. One study reported that there were no psychomimetic effects related to nebulized ketamine[28]. For benzydamine hydrochloride, numbness, burning or stinging sensation, nausea and vomiting, and dry mouth were regarded as potential adverse events. However, 3 studies demonstrated that nebulized benzydamine hydrochloride was not associated with these adverse events[11, 29, 30].

#### Sensitivity analysis

We conducted sensitivity analyses for incidence of POST at 24h operatively by excluding low quality studies(n = 7), and non 2-arm studies(n = 4). A total of 18(from 29) remained in the analysis. Overall treatment ranking and direction were not markedly affected. The SUCRA ranking were corticosteroids(0.92), magnesium(0.68), ketamine(0.55), benzydamine hydrochloride(0.44), lidocaine(0.38), and placebo(0.03). The results of node-splitting analysis did not found any inconsistency between the direct and indirect comparisons(S4 Table). And there was no significant heterogeneity across the studies(I^2^ = 1%).

## Discussion

The present network meta-analysis comprehensively analyzed 32 RCTs, which reported the occurrence of airway complaints in patients receiving 5 classes of nebulized drugs(corticosteroids, ketamine, magnesium, lidocaine, benzydamine hydrochloride) after endotracheal intubation. Overall, the direct and indirect comparison results demonstrate that, except for nebulized lidocaine, nebulized corticosteroids, ketamine, magnesium, and benzydamine hydrochloride, compared with non-analgesics, can effectively reduce the incidence of POST 24h after surgery. For early POST and remission of POST severity, only nebulized corticosteroids, ketamine, and magnesium had significant effects compared to non-analgesics. In addition, the ranking results show that corticosteroids have the best effect in almost all outcomes.

POST can be multifactorial in origin, but it is mainly associated with trauma to the larynx and the pharynx. Many factors including the size and cuff pressure of the ETT, number of suctioning attempts, the time and manipulations needs to insert the tube, female sex, younger patients, gynecological procedure, and succinylcholine administration predict POST[4].

Many studies done in past for POST including pharmacological and nonpharmacological methods. Most commonly used non-pharmacological methods are small size ETT, lubrication of tracheal cuff with water soluble jelly, gentle laryngoscopy, low intracuff pressure and smooth extubation[3]. Pharmacological methods include the use of corticosteroids, magnesium, ketamine, lidocaine, NSAIDs, and other drugs with various methods, despite their different mechanisms, ultimately produce anti-inflammatory or analgesic effects.

Although several meta-analyses in the past few years have shown that ketamine[31], lidocaine[14, 32], corticosteroids[33–35], magnesium[36, 37], benzydamine hydrochloride[38], etc. could prevent POST, there has been no comprehensive comparison between active drugs. The present review, we found that the atomized drugs except lidocaine(40mg-100mg) had similar effects to those reported in the previous reviews. The reason for the various effectiveness of lidocaine may be due to the route of administration. A meta-analysis published in 2015 by Cochrane Collaboration[32] has suggested that topical lidocaine might be more effective in preventing POST than systemic administration, but it did not analysis the effectiveness of atomized administration alone. In addition, several past studies have found that aerosolized lidocaine not only failed to relieve POST, but may even be associated with a higher incidence of POST[25, 39, 40], which may be caused by the fact that some additives in lidocaine spray may irritate tracheal mucosa[39]. Therefore, we do not recommend atomized lidocaine before intubation as a means of preventing POST.

Inhaled or aerosolized corticosteroids are commonly used in asthma treatment and are widely used in POST prophylaxis due to their ability to reduce inflammation, edema, fluid transudation, and pain severity. Our results suggest that nebulized corticosteroids are the best solution for almost all outcomes. In addition to preventing POST, cough and hoarseness, prophylactic administration of corticosteroids has been reported to reduce the incidence of postoperative laryngeal edema and reintubation[41]. The nebulized corticosteroids included in this review include dexamethasone(8mg)[10, 42, 43], budesonide(0.2mg-0.5mg)[23, 43], beclomethasone(50μg)[44, 45], mometasone[46], and fluticasone(0.5mg)[47]. We were trying to figure out which corticosteroids had the best effects. However, there is too few studies for treatment modalities and only one study[43] had reported a direct comparison between different aerosolized steroids, which might lead to misleading analysis. Therefore, further studies are needed for what kind of corticosteroids and what dose is the best approach to prevent postoperative airway complications.

In recent years, the N-methyl-D-aspartate(NMDA) receptor antagonists ketamine and magnesium have attracted attention in attenuating the symptoms of POST. NMDA has an important role in nociception and inflammation, which has been demonstrated in clinical and animal studies[48, 49]. Hence, the significant reduction in the incidence and severity of POST can be attributed to the peripheral analgesia and anti‑inflammatory effect of ketamine/magnesium nebulization. Our study suggested that although nebulized ketamine and magnesium were lower than the nebulized corticosteroids in SUCRA value of POST, there was no significant difference in the mixed comparison of ketamine, magnesium, and corticosteroids. For magnesium and ketamine, our study found almost no difference in early POST prophylaxis, but the effect of magnesium seemed to be superior to ketamine in preventing POST 24h after surgery(SUCRA value: magnesium(0.82) vs ketamine(0.55)). Similar findings have been made in gargle studies, Teymourian et al.[49] reported that the time of POST analgesia in magnesium gargle group was significantly longer than that in ketamine gargle group. And ketamine gargle only controlled pain for about 2 hours, which was also consistent in another study[50]. In addition, in terms of drug doses, the included studies used 25mg to 100mg of ketamine and 225mg to 1000mg of magnesium. Two studies compared the preventive effects of different doses of ketamine(25mg vs 50mg)[51] and magnesium(250mg vs 500mg)[52] respectively, and neither study found significant differences.

Benzydamine hydrochloride is a topical acting NSAID with local anesthetic and analgesic properties for pain relief and anti-inflammatory treatment, especially for oropharyngeal inflammation[53]. It is usually administered by gargling, atomized spraying onto the ETT cuff or oral cavity, and has been found to have therapeutic effects for POST[11, 54, 55]. However, in present study, it was found that the incidence and severity of POST did not seem to be well alleviated by oral atomization benzydamine hydrochloride. Only 24h after surgery, benzydamine hydrochloride was associated with significant reduction of incidence of POST compared with placebo, which some studies attributed to the low dose of benzydamine hydrochloride(0.75mg-1.08mg). Nevertheless, according to research reports, topical use of relatively high doses of benzydamine hydrochloride(22.5mg) is associated with a high incidence of side effects(up to 64.2%), which including local numbness, burning or stinging sensation, nausea or vomiting, etc. Although the studies we included did not report serious complications associated with nebulized benzydamine hydrochloride, the administered of nebulized benzydamine hydrochloride at high doses should be treated with caution.

A key assumption for NMA is inconsistency, which is used to assess the extent to which different sources of evidence are comparable, both substantively and statistically. However, a significant inconsistency was found in the comparison between magnesium and lidocaine in the incidence of POST 24h postoperatively. According to the theory, inconsistency include loop inconsistency and design inconsistency[56]. Based on this, we conducted a sensitivity analysis to avoid design inconsistency by excluding three-arm studies. The results demonstrated that no significant inconsistency was detected, and the overall prevention ranking and direction were not significantly affected, indicating that our results were robust.

The present review fails to fully assess all side effects related to nebulized drugs. Only 9 of 32 included studies reported intervention-related adverse events, mainly related to hemodynamics, nausea and vomiting, and numbness, and found little or no side effect from aerosolized drugs. In the included literature, Huang reported that nebulization using benzydamine hydrochloride may related to local numbness, burning, or stinging sensation. However, a recent review[38] had shown that topical use of benzydamine hydrochloride did not result in a higher risk of adverse events. Anyway, we should still use these atomized drugs with caution until their safety in POST prevention has been fully verified.

The present study has several notable strengths. First, this is the first systematic review on the efficacy and safety of applying nebulized drugs in preventing POST, providing comparisons and rankings of different active drugs. Second, we included non-English studies in our RCTs, which could improve the generalizability of our results. Third, we performed a sensitivity analysis to examine the robustness of our findings.

However, some limitations in our NWA should be mentioned. Frist, the studies included in this analysis were insufficient, especially for some important comparisons, and NWA is only a partial substitute for deficient comparisons. Second, our results were based on unadjusted estimates; more accurate outcomes would result from adjustments for other confounders such as gender, ETT size, types of surgery, and so on. Third, by the nature of meta-analysis, our results are limited by the quality of available studies. Many included studies had small sample sizes and high (or unclear) risk of bias. Fourth, we were not able to conduct a publication bias test according to the Cochrane methodology[15], because the number of studies that examined each outcome was relatively small. Thus, we cannot exclude the possibility of potential publication bias with the overestimation of our findings. Lastly, the best modality was not necessarily the most appropriate choice of medication, but also took into account economic factors and policies in different regions. Ketamine, for example, a controlled substance in many countries and can only be used under strict medical supervision because of its potential for addiction.

## Conclusions

In conclusion, the present systematic review and network meta-analysis comprehensively compared the risks of common airway complications in patients with endotracheal intubation receiving nebulized therapies. Based on the available evidence, nebulized corticosteroids, magnesium and ketamine have been shown to be effective in preventing POST, and nebulized corticosteroids appeared be the best overall modality. Additional studies with a larger sample size, better design, and more detailed outcomes are recommended to derive definitive conclusions regarding the optimal dose and risks of these therapies.

## Supporting information

S1 Table PRISMA NMA checklist. (DOCX)

S2 Table Characteristics of included studies. (DOCX)

S3 Table Simple summary data for each intervention group. (DOCX)

S4 Table Node-splitting analysis of inconsistency within network meta-analysis. p< 0.05: significant inconsistency between direct and indirect evidence.
(DOCX)

S1 Appendix Protocol for the systematic review and NMA. (PDF)

S2 Appendix Search strategy. (DOCX)

S1 Fig Risk of bias for included studies,(A) Risk of bias summary, (B) Risk of bias graph, green = low risk of bias, yellow = unclear risk of bias, red = high risk of bias.
(TIF)
