# Supplementary material for: Nebulized pharmacological agents for preventing postoperative sore throat: A systematic review and network meta-analysis
Source: PLoS One. 2020 Aug 10;15(8):e0237174. doi: 10.1371/journal.pone.0237174 (PMC7416917; doi:10.1371/journal.pone.0237174)
Supplement: S2 Table — (DOCX) [file pone.0237174.s002.docx]

**S2 Table. Characteristics of included studies**

| Study/year | Country | sample size(% female) | Mean age; years | ASA | size of ETT; mm | Cuff pressure; cmH_2_O | Endotracheal intubation technique | Mean time of surgery or anesthesia; min | Surgery | Interventions used | Administration time | Industry sponsorship/conflict of interest |
| --- | --- | --- | --- | --- | --- | --- | --- | --- | --- | --- | --- | --- |
| Almustafa/2020 | Jordan | 86(22%) | 34.5 | II，III | M：8.0 F：7.0 | Adjusted* | NR | 57(s) | Laparoscopic sleeve  gastrectomy | 1. Nebulized with 5ml solution of 8 mg dexamethasone 2. Nebulized with 5ml saline | 1h before surgery | None |
| Kamel/2020 | Egypt | 78(55%) | 31.8 | I, II | M：8.0 F：7.0 | 20-22 | NR | 120.5(s) | Elective lower abdominal and lower limb surgeries | 1. Nebulized with 5ml of magnesium sulfate 250 mg  2. Nebulized with 5ml lidocaine 2% 100mg 3. Nebulized with 5ml saline | 15min before GA. | None |
| Roy/2020 | India | 100(62%) | 29.75 | I, II | NR | 20–30 | Laryngoscopy | NR | Short duration (up to 1 hour) surgical procedures in supine position | 1. Nebulized with 5ml solution of 50mg ketamine 2. Nebulized with 5ml saline | 15min before GA | None |
| Franco-Cabrera/2019 | Mexico | 118（59%） | NR | I, II | M: 8-8.5 F: 7-7.5 | Adjusted* | Laryngoscopy/Glidescope | 126.3(a) | Otorhinolaryngologic surgery/ General surgery/Neurosurgery/Orthopedics/ Plastic surgery | 1. Nebulized with 3ml solution of 50mg ketamine 2. Nebulized with 3ml normal saline | 5min before surgery | Unclear |
| Paul/2019 | India | 120(38%) | 43.5 | I, II | M：8.0 F：7.0 | 10-20 | Laryngoscopy | 162.2(s) | Elective lumbar spine surgeries in prone position | 1. Nebulized with 0.5mg budesonide 2. Nebulized with 4ml solution of 8mg dexamethasone | 20min before GA | Unclear |
| Shahani/2019 | India | 96(54%) | 37 | I, II | M: 8.0-8.5 F: 7.0-7.5 | Adjusted* | Laryngoscopy | 90.2(s) | Elective surgical procedures in supine position | 1. Nebulized with 5ml solution of 50mg ketamine 2. Nebulized with 5ml normal saline | 30min before GA | None |
| Vaghela/2019 | India | 50(56%) | 29.5 | I, II | NR | 15-20 | Laryngoscopy | 85(s) | Elective surgeries | 1. Nebulized with 2ml solution of 50mg ketamine 2. Nebulized with 2ml solution of 4% Lignocaine | 10min before shifting to operation room | Unclear |
| Ashwini/2018 | India | 90（32%） | 37.3 | I, II | M：8.0 F：7.0 | 20-22 | Laryngoscopy | 116.3(s) | Surgeries lasting< 3h | 1. Nebulized with 5ml solution of 8mg dexamethasone 2. Nebulized with 5ml solution of MgSo4 [50% W/V 2ml] | 30min before GA | Unclear |
| Charan/2018 | India | 150(NR) | NR | I, II | M: 8-8.5 F: 7-7.5 | <20 | Laryngoscopy | NR | Surgeries in supine position lasting< 1h | 1. Nebulized with 5ml solution of 50mg ketamine 2. Nebulized with 5ml solution of 25mg ketamine 3. Nebulized with 5ml saline | 15min before surgery | None |
| Mostafa/2018 | Egypt | 90(NR) | NR | I, II | NR | <20 | NR | NR | Surgeries lasting< 2h | 1.Nebulized with 5ml solution of 250mg magnesium sulfate 2.Nebulized with 5ml solution of 25mg ketamine 3.Nebulization with 5ml saline. | before surgery | Unclear |
| Rajan/2018 | India | 46(63%) | 41.9 | I, II | M：8.0 F：7.0 | 20–22 | Laryngoscopy | 184.2(s) | Laparoscopic surgeries (laparoscopic sterilisation and  diagnostic laparoscopy) lasting <2h | 1.Nebulized with 200μg budesonide 2.no treatment | 10min before intubation, repeated 6h after extubation | None |
| Segaran/2018 | India | 80(53%) | 34.5 | I, II | M: 8.0-8.5 F: 7.0-7.5 | 20 | Laryngoscopy | NR | Elective surgeries | 1. Nebulized with 5ml solution of 50mg ketamine 2. Nebulized with 5ml solution of 250 mg magnesium sulfate | 15min before GA | None |
| Shah/2018 | India | 200(35%) | 33.5 | I, II | M: 8.0 F: 7.0 | 20-22 | Laryngoscopy | 65(s) | Surgeries in supine position lasting< 2h | 1.Nebulized with 3ml solution of 300mg magnesium sulfate 2.Nebulized with 3ml of saline | before GA | None |
| Thomas/2018 | India | 96(54%) | 37 | I, II | M: 8.0-8.5 F: 7.0-7.5 | Adjusted* | Laryngoscopy | 90.1(s) | Surgeries in supine position lasting< 2h | 1. Nebulized with 5ml solution of 50mg ketamine 2. Nebulized with 5ml saline | 15min before GA | None |
| Aditya/2017 | India | 50(30%) | NR | I, II | NR | NR | Laryngoscopy | NR | Surgeries lasting< 1h | 1. Nebulized with 5ml solution of 50mg ketamine 2. Nebulized with 5ml normal saline | before GA | Unclear |
| Jain/2017 | India | 150(100%) | NR | I, II | F: 7.0-7.5 | < 20 | Laryngoscopy | NR | Laparoscopic cholecystectomy | 1. Nebulized with 3ml solution of 225mg magnesium sulfate 2.Nebulized with 3ml solution of 50mg ketamine 3. Nebulized with 3ml saline | 15min before GA | None |
| Rajan/2017 | India | 60(NR) | NR | I–III | M: 8.0-8.5 F: 7.0-7.5 | 20-25 | Laryngoscopy | NR | Elective abdominal and lower limb surgeries | 1. Nebulized with 5ml solution of 50mg ketamine 2. Nebulized with 5ml solution of 250 mg magnesium sulfate 3. Nebulized with 5ml solution of 500mg magnesium sulfate 4.Nebulized with 5ml saline | 15min before surgery | None |
| Sharma/2017 | India | 140(30%) | 38 | I, II | M: 8.0-8.5 F: 7.0-7.5 | 20(supine)/10-20(prone) | Laryngoscopy | 129.5(s) | Lumbar spine surgery | 1. Nebulized with 5ml solution of 225mg magnesium sulfate 2. Nebulized with 5ml saline | 20min before GA | Unclear |
| Ayatollahi/2016 | Iran | 68（49%） | 31.15 | I, II | NR | 20-25 | Laryngoscopy | NR | Surgeries lasting between 0.5-1.5h | 1. Nebulized with 2ml solution of 25mg ketamine 2. Nebulized with 2ml saline | before intubation | None |
| Yadav/2016 | India | 100(47%) | 40.9 | I, II | M: 8.0 F: 7.0 | 20 | NR | NR | Surgeries lasting> 2h | 1. Nebulized with 3ml solution of 225mg magnesium sulfate 2. Nebulized with 3ml saline | 20min before GA | Unclear |
| Ahuja/2015 | India | 100 (34%) | 41.4 | I, II | M: 8-8.5 F: 7-7.5 | Adjusted* | NR | 54.5(s) | Surgeries in supine position lasting< 1h | 1. Nebulized with 5ml solution of 50mg ketamine 2. Nebulized with 5ml saline | 25min before GA | None |
| Bashir/2014 | Pakistan | 200(38.9%) | 32.9 | I, II | NR | NR | NR | 89.1(s) | Surgeries lasting> 1h | 1.Inhalation with beclomethasone (one puff) 2. No treatment | Before surgery | None |
| Narasethakamo/2011 | Thailand | 42(NR) | 50.8 | I, II | M: 8.0 F: 7.5 | 25 | Laryngoscopy | 97.8(a) | Surgeries in supine position | 1.Sprayed with 0.05% mometasone furoate solution  2. Sprayed with saline | Before intubation | None |
| Tazeh-Kand/2010 | Iran | 120(100%) | 26.5 | I, II | F: 7.0 | < 20 | Laryngoscopy | 53.9(a)/40.3(s) | Elective cesarean delivery under GA | 1.Inhalation with 500μg fluticasone 2.no treatment | After arrival in the operating room | Unclear |
| Yang/2010 | China | 80(55%) | 47.9 | I, II | M: 7.5 F: 7.0 | 25 | NR | 151.2(a) | Surgeries in supine position | 1. Sprayed with ketamine 0.2 mg/kg（2ml） 2. Sprayed with 2ml saline | 30sec before intubation | Unclear |
| Honarmand/2008 | Iran | 120(38%) | 32.3 | I, II | M:7.5 F:7.0 | < 20 | Laryngoscopy | 103.7(s) | Gynecological, abdominal, and orthopedic surgeries in the supine position with expected extubation immediately after the operation | 1.Intravenous lidocaine 1mg/kg  2. Intravenous lidocaine 1.5 mg/kg  3. Sprayed with 50μg beclomethasone 4.Intravenous saline | Before intubation | None |
| Maruyama/2004 | Japan | 168(51%) | 59.1 | I–III | M：8.0-9.0 F：7.0-8.0 | < 20 | Laryngoscopy | 149.6(a) | Surgeries in supine position | 1. Sprayed with lidocaine 5 times  2. Sprayed with lidocaine 10 times 3.Sprayed with 1ml saline | Before intubation | Unclear |
| Soltani/2002 | Iran | 204(42.1%) | 63 | I, II | M: 8.0-8.5 F: 7.0-7.5 | NR | Laryngoscopy | 45(s) | Cataract extraction | 1.Sprayed with 10% lidocaine (ETT) 2. Sprayed with 10% lidocaine(OC) 3.Lubricated with 2.5g of 2% lidocaine jelly 4.Intravenous 1.5 mg/kg lidocaine 5.Sprayed with 7 to 8ml of 2% lidocaine(ETT) 6.lubricated with normal saline(ETT) | Before intubation | Unclear |
| Dogan/2004 | Turkey | 80 (50%) | NR | I, II | M: 9.0 F: 8.0 | NR | NR | NR | NR | 1.Sprayed with 1.08 mg benzydamine (four puffs) 2. Sprayed with Saline (four puffs) | Before intubation | Unclear |
| Gulhas/2007 | Turkey | 180 (74.4%) | 36.1 | I, II | M: 8.0 F: 7.0 | 20–25 | Laryngoscopy | 114.2(s) | Elective surgeries | 1.Sprayed with benzydamine(four puffs) 2.two pastilles of dexpanthenol 3.Sprayed with distilled water(four puffs) | 30min before the surgery | Unclear |
| Huang/2010 | China | 378 (51.9%) | 47.6 | NR | M: 7.0 F: 6.5 | 20–25 | Laryngoscopy | 178(a) | Colon rectal surgery/General surgery/Genitourinary surgery/Gynecologic surgery /Ophthalmologic surgery /Orthopedic surgery/Plastic surgery | 1. Sprayed with 0.75 mg of 0.15% benzydamine(OC)+0.5ml distilled water(ETT) 2.Sprayed with 0.75 mg of 0.15% benzydamine(OC+ETT) 3.Sprayed with 0.75 mg 0.15% benzydamine(ETT)+0.5ml distilled water(OC) 4.Sprayed with 0.5ml distilled water(OC+ETT) | 5min before GA | None |
| Chang/2015 | South Korea | 92 (33.7%) | 55.5 | I–III | Double-lumen M: 37 Fr F:35 Fr | 20 | Laryngoscopy | 175.5(a)/128(s) | Thoracic surgery | 1.Sparyed with 0.9 mg of 0.3% benzydamine(three puffs) 2.Sparyed with saline(three puffs) | Before intubation | None |
| ASA, The American Society of Anesthesiologists physical status classification system; ETT, endotracheal tube; F, female; M, male; adjusted*, ETT cuff was inflated with air until no air leakage was audible; NR, not reported; GA, general anesthesia; Fr, French gauge; OC, oral cavity. | | | | | | | | | | | | |
