# Supplementary material for: Nebulized pharmacological agents for preventing postoperative sore throat: A systematic review and network meta-analysis
Source: PLoS One. 2020 Aug 10;15(8):e0237174. doi: 10.1371/journal.pone.0237174 (PMC7416917; doi:10.1371/journal.pone.0237174)
Supplement: S3 Table — (DOCX) [file pone.0237174.s003.docx]

**S3 Table. Simple summary data for each intervention group**

**1. Incidence of POST 24h after extubation**

| ID | Study | Year | Treatment | Responders | Sample size |
| --- | --- | --- | --- | --- | --- |
| 1 | Yadav[1] | 2016 | Magnesium | 0 | 50 |
|  |  |  | Placebo | 7 | 50 |
| 2 | Thomas[2] | 2018 | Ketamine | 4 | 48 |
|  |  |  | Placebo | 11 | 48 |
| 3 | Tazeh-kand[3] | 2010 | Corticosteroids | 8 | 60 |
|  |  |  | Placebo | 24 | 60 |
| 4 | Segaran[4] | 2018 | Magnesium | 0 | 40 |
|  |  |  | Ketamine | 0 | 40 |
| 5 | Rajan[5] | 2018 | Corticosteroids | 0 | 23 |
|  |  |  | Placebo | 16 | 23 |
| 6 | Rajan[6] | 2017 | Placebo | 6 | 15 |
|  |  |  | Ketamine | 0 | 15 |
|  |  |  | Magnesium | 1 | 30 |
| 7 | Kamel[7] | 2020 | Magnesium | 7 | 26 |
|  |  |  | Lidocaine | 24 | 26 |
|  |  |  | Placebo | 24 | 26 |
| 8 | Charan[8] | 2018 | Ketamine | 4 | 100 |
|  |  |  | Placebo | 7 | 50 |
| 9 | Almustafa[9] | 2019 | Corticosteroids | 11 | 40 |
|  |  |  | Placebo | 35 | 40 |
| 10 | Ahuja[10] | 2016 | Placebo | 7 | 50 |
|  |  |  | Ketamine | 2 | 50 |
| 11 | Vaghela[11] | 2019 | Ketamine | 0 | 25 |
|  |  |  | Lidocaine | 0 | 25 |
| 12 | Shahani[12] | 2019 | Ketamine | 4 | 48 |
|  |  |  | Placebo | 11 | 48 |
| 13 | Roy[13] | 2016 | Ketamine | 2 | 50 |
|  |  |  | Placebo | 18 | 50 |
| 14 | Mostafa[14] | 2018 | Magnesium | 0 | 30 |
|  |  |  | Ketamine | 0 | 30 |
|  |  |  | Placebo | 3 | 30 |
| 15 | Jain[15] | 2017 | Ketamine | 2 | 50 |
|  |  |  | Magnesium | 2 | 50 |
|  |  |  | Placebo | 7 | 50 |
| 16 | Shah[16] | 2018 | Magnesium | 10 | 100 |
|  |  |  | Placebo | 25 | 100 |
| 17 | Narasethakamol[17] | 2011 | Corticosteroids | 2 | 20 |
|  |  |  | Placebo | 8 | 20 |
| 18 | Soltani[18] | 2002 | Lidocaine | 4 | 34 |
|  |  |  | Placebo | 8 | 34 |
| 19 | Honarmand[19] | 2008 | Corticosteroids | 3 | 30 |
|  |  |  | Placebo | 14 | 30 |
| 20 | Ayatollahi[20] | 2016 | Ketamine | 17 | 34 |
|  |  |  | Placebo | 21 | 34 |
| 21 | Bashir[21] | 2014 | Corticosteroids | 0 | 100 |
|  |  |  | Placebo | 1 | 100 |
| 22 | Sharma[22] | 2017 | Magnesium | 5 | 70 |
|  |  |  | Placebo | 18 | 70 |
| 23 | Ashwini[23] | 2018 | Magnesium | 1 | 40 |
|  |  |  | Corticosteroids | 1 | 40 |
| 24 | Yang[24] | 2010 | Ketamine | 5 | 40 |
|  |  |  | Placebo | 13 | 40 |
| 25 | Aditya[25] | 2017 | Ketamine | 0 | 25 |
|  |  |  | Placebo | 3 | 25 |
| 26 | Dogan[26] | 2004 | Benzydamine hydrochloride | 2 | 40 |
|  |  |  | Placebo | 12 | 40 |
| 27 | Gulhas[27] | 2007 | Benzydamine hydrochloride | 7 | 60 |
|  |  |  | Placebo | 16 | 60 |
| 28 | Huang[28] | 2010 | Benzydamine hydrochloride | 10 | 95 |
|  |  |  | Placebo | 22 | 94 |
| 29 | Chang[29] | 2015 | Benzydamine hydrochloride | 8 | 46 |
|  |  |  | Placebo | 21 | 46 |

**2. Incidence of POST 1h after extubation**

| ID | Study | Year | Treatment | Responders | Sample size |
| --- | --- | --- | --- | --- | --- |
| 1 | Yadav[1] | 2016 | Magnesium | 7 | 50 |
|  |  |  | Placebo | 11 | 50 |
| 2 | Thomas[2] | 2018 | Ketamine | 6 | 48 |
|  |  |  | Placebo | 16 | 48 |
| 3 | Tazeh-kand[3] | 2010 | Corticosteroids | 2 | 60 |
|  |  |  | Placebo | 22 | 60 |
| 4 | Segaran[4] | 2018 | Magnesium | 4 | 40 |
|  |  |  | Ketamine | 2 | 40 |
| 5 | Rajan[5] | 2018 | Corticosteroids | 6 | 23 |
|  |  |  | Placebo | 19 | 23 |
| 6 | Rajan[6] | 2017 | Placebo | 15 | 15 |
|  |  |  | Ketamine | 2 | 15 |
|  |  |  | Magnesium | 20 | 30 |
| 7 | Kamel[7] | 2020 | Magnesium | 6 | 26 |
|  |  |  | Lidocaine | 8 | 26 |
|  |  |  | Placebo | 26 | 26 |
| 8 | Charan[8] | 2018 | Ketamine | 6 | 100 |
|  |  |  | Placebo | 13 | 50 |
| 9 | Ahuja[10] | 2016 | Placebo | 13 | 50 |
|  |  |  | Ketamine | 3 | 50 |
| 10 | Vaghela[11] | 2019 | Ketamine | 5 | 25 |
|  |  |  | Lidocaine | 12 | 25 |
| 11 | Shahani[12] | 2019 | Ketamine | 6 | 48 |
|  |  |  | Placebo | 16 | 48 |
| 12 | Roy[13] | 2016 | Ketamine | 6 | 50 |
|  |  |  | Placebo | 19 | 50 |
| 13 | Mostafa[14] | 2018 | Magnesium | 11 | 30 |
|  |  |  | Ketamine | 20 | 30 |
|  |  |  | Placebo | 18 | 30 |
| 14 | Jain[15] | 2017 | Ketamine | 3 | 50 |
|  |  |  | Magnesium | 4 | 50 |
|  |  |  | Placebo | 11 | 50 |
| 15 | Shah[16] | 2018 | Magnesium | 42 | 100 |
|  |  |  | Placebo | 58 | 100 |
| 16 | Narasethakamol[17] | 2011 | Corticosteroids | 8 | 20 |
|  |  |  | Placebo | 15 | 20 |
| 17 | Soltani[18] | 2002 | Lidocaine | 26 | 34 |
|  |  |  | Placebo | 26 | 34 |
| 18 | Honarmand[19] | 2008 | Corticosteroids | 6 | 30 |
|  |  |  | Placebo | 15 | 30 |
| 19 | Ayatollahi[20] | 2016 | Ketamine | 26 | 34 |
|  |  |  | Placebo | 31 | 34 |
| 20 | Sharma[22] | 2017 | Magnesium | 10 | 70 |
|  |  |  | Placebo | 49 | 70 |
| 21 | Ashwini[23] | 2018 | Magnesium | 16 | 40 |
|  |  |  | Corticosteroids | 7 | 40 |
| 22 | Yang[24] | 2010 | Ketamine | 10 | 40 |
|  |  |  | Placebo | 18 | 40 |
| 23 | Aditya[25] | 2017 | Ketamine | 3 | 25 |
|  |  |  | Placebo | 6 | 25 |
| 24 | Franco-Cabrera[30] | 2019 | Ketamine | 24 | 58 |
|  |  |  | Placebo | 20 | 54 |
| 25 | Maruyama[31] | 2004 | Lidocaine | 48 | 93 |
|  |  |  | Placebo | 13 | 47 |
| 26 | Gulhas[27] | 2007 | Benzydamine hydrochloride | 29 | 60 |
|  |  |  | Placebo | 35 | 60 |
| 27 | Huang[28] | 2010 | Benzydamine hydrochloride | 22 | 95 |
|  |  |  | Placebo | 38 | 94 |
| 28 | Chang[29] | 2015 | Benzydamine hydrochloride | 10 | 46 |
|  |  |  | Placebo | 23 | 46 |

**3. Incidence of moderate to severe POST 24h after extubation**

| ID | Study | Year | Treatment | Responders | Sample size |
| --- | --- | --- | --- | --- | --- |
| 1 | Thomas[2] | 2018 | Ketamine | 0 | 48 |
|  |  |  | Placebo | 1 | 48 |
| 2 | Tazeh-kand[3] | 2010 | Corticosteroids | 2 | 60 |
|  |  |  | Placebo | 9 | 60 |
| 3 | Segaran[4] | 2018 | Magnesium | 0 | 40 |
|  |  |  | Ketamine | 0 | 40 |
| 4 | Rajan[5] | 2018 | Corticosteroids | 0 | 23 |
|  |  |  | Placebo | 7 | 23 |
| 5 | Rajan[6] | 2017 | Placebo | 1 | 15 |
|  |  |  | Ketamine | 0 | 15 |
|  |  |  | Magnesium | 0 | 30 |
| 6 | Kamel[7] | 2020 | Magnesium | 0 | 26 |
|  |  |  | Lidocaine | 9 | 26 |
|  |  |  | Placebo | 10 | 26 |
| 7 | Charan[8] | 2018 | Ketamine | 0 | 100 |
|  |  |  | Placebo | 2 | 50 |
| 8 | Ahuja[10] | 2016 | Placebo | 2 | 50 |
|  |  |  | Ketamine | 0 | 50 |
| 9 | Shahani[12] | 2019 | Ketamine | 0 | 48 |
|  |  |  | Placebo | 1 | 48 |
| 10 | Roy[13] | 2016 | Ketamine | 0 | 50 |
|  |  |  | Placebo | 11 | 50 |
| 11 | Mostafa[14] | 2018 | Magnesium | 0 | 30 |
|  |  |  | Ketamine | 0 | 30 |
|  |  |  | Placebo | 0 | 30 |
| 12 | Jain[15] | 2017 | Ketamine | 0 | 50 |
|  |  |  | Magnesium | 0 | 50 |
|  |  |  | Placebo | 3 | 50 |
| 13 | Shah[16] | 2018 | Magnesium | 4 | 100 |
|  |  |  | Placebo | 10 | 100 |
| 14 | Narasethakamol[17] | 2011 | Corticosteroids | 0 | 20 |
|  |  |  | Placebo | 2 | 20 |
| 15 | Ayatollahi[20] | 2016 | Ketamine | 4 | 34 |
|  |  |  | Placebo | 1 | 34 |
| 16 | Sharma[22] | 2017 | Magnesium | 1 | 70 |
|  |  |  | Placebo | 10 | 70 |
| 17 | Ashwini[23] | 2018 | Magnesium | 0 | 40 |
|  |  |  | Corticosteroids | 0 | 40 |
| 18 | Yang[24] | 2010 | Ketamine | 0 | 40 |
|  |  |  | Placebo | 5 | 40 |
| 19 | Huang[28] | 2010 | Benzydamine hydrochloride | 6 | 95 |
|  |  |  | Placebo | 17 | 94 |

**4. Incidence of postoperative cough 24h after extubation**

| ID | Study | Year | Treatment | Responders | Sample size |
| --- | --- | --- | --- | --- | --- |
| 1 | Tazeh-kand[3] | 2010 | Corticosteroids | 6 | 60 |
|  |  |  | Placebo | 25 | 60 |
| 2 | Rajan[5] | 2018 | Corticosteroids | 1 | 23 |
|  |  |  | Placebo | 4 | 23 |
| 3 | Rajan[6] | 2017 | Placebo | 0 | 15 |
|  |  |  | Ketamine | 0 | 15 |
|  |  |  | Magnesium | 0 | 30 |
| 4 | Vaghela[11] | 2019 | Ketamine | 2 | 25 |
|  |  |  | Lidocaine | 0 | 25 |
| 5 | Honarmand[19] | 2008 | Corticosteroids | 2 | 30 |
|  |  |  | Placebo | 12 | 30 |
| 6 | Ashwini[23] | 2018 | Magnesium | 0 | 40 |
|  |  |  | Corticosteroids | 0 | 40 |
| 7 | Huang[28] | 2010 | Benzydamine hydrochloride | 19 | 95 |
|  |  |  | Placebo | 18 | 94 |

**5. Incidence of postoperative hoarseness 24h after extubation**

| ID | Study | Year | Treatment | Responders | Sample size |
| --- | --- | --- | --- | --- | --- |
| 1 | Tazeh-kand[3] | 2010 | Corticosteroids | 15 | 60 |
|  |  |  | Placebo | 30 | 60 |
| 2 | Rajan[5] | 2018 | Corticosteroids | 0 | 23 |
|  |  |  | Placebo | 2 | 23 |
| 3 | Rajan[6] | 2017 | Placebo | 0 | 15 |
|  |  |  | Ketamine | 0 | 15 |
|  |  |  | Magnesium | 0 | 30 |
| 4 | Vaghela[11] | 2019 | Ketamine | 0 | 25 |
|  |  |  | Lidocaine | 0 | 25 |
| 5 | Honarmand[19] | 2008 | Corticosteroids | 3 | 30 |
|  |  |  | Placebo | 8 | 30 |
| 6 | Ayatollahi[20] | 2016 | Ketamine | 5 | 34 |
|  |  |  | Placebo | 5 | 34 |
| 7 | Ashwini[23] | 2018 | Magnesium | 0 | 40 |
|  |  |  | Corticosteroids | 0 | 40 |
| 8 | Huang[28] | 2010 | Benzydamine hydrochloride | 4 | 95 |
|  |  |  | Placebo | 5 | 94 |

1. Yadav M, Chalumuru N, Gopinath R. Effect of magnesium sulfate nebulization on the incidence of postoperative sore throat. Journal of anaesthesiology, clinical pharmacology. 2016;32(2):168-71. Epub 2016/06/09. doi: 10.4103/0970-9185.173367. PubMed PMID: 27275043.

2. Thomas D, Bejoy R, Zabrin N, Beevi S. Preoperative ketamine nebulization attenuates the incidence and severity of postoperative sore throat: A randomized controlled clinical trial. Saudi journal of anaesthesia. 2018;12(3):440-5. Epub 2018/08/14. doi: 10.4103/sja.SJA_47_18. PubMed PMID: 30100844.

3. Tazeh-Kand NF, Eslami B, Mohammadian K. Inhaled fluticasone propionate reduces postoperative sore throat, cough, and hoarseness. Anesthesia and analgesia. 2010;111(4):895-8. Epub 2010/03/20. doi: 10.1213/ANE.0b013e3181c8a5a2. PubMed PMID: 20237046.

4. Segaran S, Bacthavasalame AT, Venkatesh RR, Zachariah M, George SK, Kandasamy R. Comparison of Nebulized Ketamine with Nebulized Magnesium Sulfate on the Incidence of Postoperative Sore Throat. Anesthesia, essays and researches. 2018;12(4):885-90. Epub 2019/01/22. doi: 10.4103/aer.AER_148_18. PubMed PMID: 30662125.

5. Rajan S, Tosh P, Paul J, Kumar L. Effect of inhaled budesonide suspension, administered using a metered dose inhaler, on post-operative sore throat, hoarseness of voice and cough. Indian journal of anaesthesia. 2018;62(1):66-71. doi: 10.4103/ija.IJA_382_17. PubMed PMID: 29416153.

6. Rajan S, Malayil GJ, Varghese R, Kumar L. Comparison of Usefulness of Ketamine and Magnesium Sulfate Nebulizations for Attenuating Postoperative Sore Throat, Hoarseness of Voice, and Cough. Anesthesia, essays and researches. 2017;11(2):287-93. Epub 2017/07/01. doi: 10.4103/0259-1162.181427. PubMed PMID: 28663608.

7. Kamel AAF, Ibrahem Amin OA. The Effect of preoperative nebulized: Magnesium sulfate versus lidocaine on the prevention of post-intubation sore throat. Egyptian Journal of Anaesthesia. 2020;36(1):1-6. doi: 10.1080/11101849.2020.1723330.

8. Charan SD, Khilji MY, Jain R, Devra V, Saxena M. Inhalation of Ketamine in Different Doses to Decrease the Severity of Postoperative Sore Throat in Surgeries under General Anesthesia Patients. Anesthesia, essays and researches. 2018;12(3):625-9. Epub 2018/10/05. doi: 10.4103/aer.AER_65_18. PubMed PMID: 30283166.

9. Almustafa M, Obeidat F, Mismar A, Rashdan M, Jabaiti K, Alryalat SA, et al. Role of Preoperative Dexamethasone Nebulization in Reducing Bougie Complications Encountered After Sleeve Gastrectomy: a Prospective Double-Blind Control Interventional Study. Obesity surgery. 2020;30(2):501-6. doi: 10.1007/s11695-019-04202-x. PubMed PMID: 31646475.

10. Ahuja V, Mitra S, Sarna R. Nebulized ketamine decreases incidence and severity of post-operative sore throat. Indian journal of anaesthesia. 2015;59(1):37-42. Epub 2015/02/17. doi: 10.4103/0019-5049.149448. PubMed PMID: 25684812.

11. Vaghela AP, Shah RS, Shah DV. Comparative Evaluation of Incidence of Post-operative Sore Throat after Nebulization with Ketamine and Lignocaine in Patients Under-going General Anaesthesia. National Journal of Medical Research. 2019;9(4):168-70.

12. Shahani J, Meena SJIJoSR. A RANDOMIZED CONTROLLED STUDY OF REDUCTION IN SEVERITY OF POSTOPERATIVE SORETHROAT USING PREOPERATIVE KETAMINE NEBULIZATION. International Journal of Scientific Research. 2019;8(1).

13. Roy A, Biswas C, Bhattacharjee DP. NEBULIZATION WITH KETAMINE ATTENUATES POST OPERATIVE SORE THROAT AFTER OROTRACHEAL INTUBATION-A RANDOMIZED, PLACEBO CONTROLLED, SINGLE BLIND CLINICAL TRIAL. Indian Journal of Applied Research. 2020;9(12):20-4.

14. Mostafa EA, Abdel Rahman AS, Mahmoud MDJTEJoHM. Magnesium Sulfate Nebulizer versus Ketamine Nebulizer in Decreasing Incidence and Severity of Post Operative Sore Throat with Endotracheal Intubation in Adults. 2018;73(8):7244-50.

15. Jain S, Barasker SK. A comparative study of preoperative ketamine and MgSO4 nebulisation for incidence of post operative sore throat after endotracheal intubation. Int J Contemp Med Res. 2017;4:1356-9.

16. Shah DR, Shah SM, Parikh TP, Reshamwala NS, Chavda AJJA. ROLE OF MAGNESIUM SULFATE NEBULISATION ON REDUCING THE INCIDENCE OF POST-OPERATIVE SORE THROAT (POST). 2018;34(10):32-12. doi: 10.14260/jemds/2018/892.

17. Narasethakamol A, Techanivate A, Saothongthong J, Yurakate N, Cousnit P. Application of mometasone spray to reduce sore throat after tracheal intubation. Journal of the Medical Association of Thailand = Chotmaihet thangphaet. 2011;94(8):958-64. Epub 2011/08/26. PubMed PMID: 21863678.

18. Soltani HA, Aghadavoudi O. The effect of different lidocaine application methods on postoperative cough and sore throat. Journal of clinical anesthesia. 2002;14(1):15-8. doi: 10.1016/S0952-8180(01)00344-0.

19. Honarmand A, Safavi M. Beclomethasone inhaler versus intravenous lidocaine in the prevention of postoperative airway and throat complaints: a randomized, controlled trial. Annals of Saudi medicine. 2008;28(1):11-6. Epub 2008/02/27. doi: 10.5144/0256-4947.2008.11. PubMed PMID: 18299653.

20. Ayatollahi V, Moslemin F, Behdad S, Hatami M. Effect of Ketamine Spraying on the Postoperative Sore Throat and Hoarseness after Tracheal Intubation. British Journal of Medicine & Medical Research. 2016;17:1-7. doi: 10.9734/BJMMR/2016/26873

21. Bashir I, Masood N. Prophylaxis of postintubation sore throat by the use of single puff inhalation of beclomethasone dipropionate preoperatively. Pakistan Armed Forces Medical Journal. 2014;64(1):145-9.

22. Sharma M, Goyal MK, Purohit S, Maniyar F, Gupta D. Comparison of magnesium sulfate and normal saline (placebo) nebulization for prevention of postoperative sore throat in patients undergoing lumbar spine surgeries under general anaesthesia with endotracheal intubation in prone position. Int J Sci Res. 2017;6:656-8.

23. Ashwini H, Seema Kumari K, Lavanya R. Comparative study of dexamethasone nebulisation with magnesium sulphate nebulisation in preventing post operative sore throat following endotracheal intubation. Indian Journal of Clinical Anaesthesia. 2018;5(3):341-7. doi: 10.18231/2394-4994.2018.0065.

24. Yang W, Wang Y, Li Y-h, Shao J-l. Clinical Observation of Ketamine for Attenuating Postoperative Sore Throat. Journal of Kunming Medical University. 2010;4:166-19.

25. Aditya AK, Das B, Mishra DK. Assessment of nebulized ketamine for reductions of incidence and severity of post-operative sore throat. Int J Med Health Res. 2017;3:130-2.

26. Nazım Doğan ZUS, Hüsnü Kürşad, Mehmet Kızılkaya. Topikal Olarak Uygulanan Benzidamin Hidroklorür'ün Entübasyona Bağlı Postoperatif Boğaz Ağrısı Üzerine Etkisi. Türk Anesteziyoloji ve Reanimasyon Derneği Dergisi. 2004;32(1):22-6.

27. Gulhas N, Canpolat H, Cicek M, Yologlu S, Togal T, Durmus M, et al. Dexpanthenol pastille and benzydamine hydrochloride spray for the prevention of post‐operative sore throat. Acta anaesthesiologica Scandinavica. 2007;51(2):239-43. doi: 10.1111/j.1399-6576.2006.01180.x. PubMed PMID: 17073853.

28. Huang YS, Hung NK, Lee MS, Kuo CP, Yu JC, Huang GS, et al. The effectiveness of benzydamine hydrochloride spraying on the endotracheal tube cuff or oral mucosa for postoperative sore throat. Anesthesia and analgesia. 2010;111(4):887-91. doi: 10.1213/ANE.0b013e3181e6d82a. PubMed PMID: 20581162.

29. Chang JE, Min SW, Kim CS, Han SH, Kwon YS, Hwang JY. Effect of prophylactic benzydamine hydrochloride on postoperative sore throat and hoarseness after tracheal intubation using a double-lumen endobronchial tube: a randomized controlled trial. Canadian journal of anaesthesia = Journal canadien d'anesthesie. 2015;62(10):1097-103. Epub 2015/07/08. doi: 10.1007/s12630-015-0432-x. PubMed PMID: 26149601.

30. Franco-Cabrera M, Aguirre-Ibarra CP, Nava-López JA, Méndez-Hernández AZ, Duarte-Pérez KJ, Vargas-Aguilar DM, et al. Ketamina nebulizada para la prevención del dolor faríngeo postoperatorio. 2019;42(1):7-18.

31. Maruyama K, Sakai H, Miyazawa H, Iijima K, Toda N, Kawahara S, et al. Laryngotracheal application of lidocaine spray increases the incidence of postoperative sore throat after total intravenous anesthesia. Journal of anesthesia. 2004;18(4):237-40. Epub 2004/11/19. doi: 10.1007/s00540-004-0264-2. PubMed PMID: 15549464.
