# Supplementary material for: Nebulized pharmacological agents for preventing postoperative sore throat: A systematic review and network meta-analysis
Source: PLoS One. 2020 Aug 10;15(8):e0237174. doi: 10.1371/journal.pone.0237174 (PMC7416917; doi:10.1371/journal.pone.0237174)
Supplement: S4 Table — p< 0.05: significant inconsistency between direct and indirect evidence. (DOCX) [file pone.0237174.s004.docx]

**S4 Table. Node-splitting analysis of inconsistency within network meta-analysis.**

| Comparsion | p-value | OR(95%CI) |
| --- | --- | --- |
| incidence of POST 24h after surgery | | |
| Magnesium VS Corticosteroids |  |  |
| direct | 0.83442 | 0.98 (0.020, 48) |
| indirect |  | 1.4 (0.44, 4.6) |
| network |  | 1.4 (0.45, 4.1) |
| Placebo VS Corticosteroids |  |  |
| direct | 0.83327 | 12. (5.3, 32.) |
| indirect |  | 8.3 (0.16, 4.3e+02) |
| network |  | 12. (5.4, 29.) |
| Magnesium VS Ketamine |  |  |
| direct | 0.28766 | 1.3 (0.21, 9.3) |
| indirect |  | 0.43 (0.13, 1.3) |
| network |  | 0.54 (0.21, 1.3) |
| Lidocaine VS Ketamine |  |  |
| direct | 0.41155 | 0.98 (0.022, 51.) |
| indirect |  | 4.9 (1.2, 23.) |
| network |  | 3.9 (1.0, 15.) |
| Placebo VS Ketamine |  |  |
| direct | 0.76734 | 4.8 (2.6, 10.) |
| indirect |  | 3.2 (0.19, 55.) |
| network |  | 4.6 (2.7, 9.2) |
| Lidocaine VS Magnesium |  |  |
| direct | 0.02005 | 41. (6.5, 4.2e+02) |
| indirect |  | 2.0 (0.39，11.) |
| network |  | 7.1 ( 2., 28.) |
| incidence of POST 1h after surgery | | |
| Magnesium VS Corticosteroids |  |  |
| direct | 0.78202 | 3.3 (0.27. 40.) |
| indirect |  | 2.2 (0.45, 11.) |
| network |  | 2.5 (0.67, 9.) |
| Placebo VS Corticosteroids |  |  |
| direct | 0.77929 | 8.6 (2.3, 33.) |
| indirect |  | 13. (0.96, 1.9e+02) |
| network |  | 9.9 (3.0, 30.) |
| Magnesium VS Ketamine |  |  |
| direct | 0.36547 | 1.7 (0.46, 6.5) |
| indirect |  | 0.74 (0.19, 2.7) |
| network |  | 1.1 (0.43, 2.7) |
| Lidocaine VS Ketamine |  |  |
| direct | 0.72745 | 3.9 (0.30, 53.) |
| indirect |  | 2.3 (0.50, 11.) |
| network |  | 2.7 (0.73, 9.6) |
| Placebo VS Ketamine |  |  |
| direct | 0.46486 | 3.7 (1.7, 8.3) |
| indirect |  | 8.6 (0.95, 92.) |
| network |  | 4.0 (2.1, 8.2) |
| Lidocaine VS Magnesium |  |  |
| direct | 0.31824 | 1.5(0.21, 11.) |
| indirect |  | 4.9 (1.4, 17.) |
| network |  | 2.5 (0.64, 9.4) |
| incidence of moderate to severe POST 24h after surgery | | |
| Magnesium VS Corticosteroids |  |  |
| direct | 0.81663 | 1.0 (0.0021, 5.0e+02) |
| indirect |  | 2.3 (0.025, 3.7e+02) |
| network |  | 1.6 (0.048, 65.) |
| Placebo VS Corticosteroids |  |  |
| direct | 0.82545 | 48.(1.4, 5.9e+03) |
| indirect |  | 23. (0.035, 2.5e+04) |
| network |  | 35. (1 .9, 1.7e+03) |
| Magnesium VS Ketamine |  |  |
| direct | 0.78501 | 0.93 (0.012, 69.) |
| indirect |  | 2.0 (0.034, 2.9e+02) |
| network |  | 1.3 (0.090, 26.) |
| Placebo VS Ketamine |  |  |
| direct | 0.902 | 34. (3.4, 9.0e+02) |
| indirect |  | 22. (0.023, 3.2e+04) |
| network |  | 28. (3.6, 5.9e+02) |
| Placebo VS Magnesium |  |  |
| direct | 0.71327 | 19. (1.6, 4.2e+02) |
| indirect |  | 54. (0.43, 1.6e+04) |
| network |  | 22. (2.9, 3.3e+02) |
| Sensitivity analysis | | |
| Magnesium VS Corticosteroids |  |  |
| direct | 0.75128 | 1.0 (0.024, 51.) |
| indirect |  | 1.8 (0.42, 6.5) |
| network |  | 1.7 (0.47, 5.4) |
| Placebo VS Corticosteroids |  |  |
| direct | 0.76182 | 8.3 (3.8, 19.) |
| indirect |  | 4.8 (0.094, 2.4e+02) |
| network |  | 8. (3.8, 18.) |
| Magnesium VS Ketamine |  |  |
| direct | 0.87633 | 0.99 (0.025, 47.) |
| indirect |  | 0.77 (0.19, 2.4) |
| network |  | 0.82 (0.23, 2.3) |
| Placebo VS Ketamine |  |  |
| direct | 0.90377 | 3.8 (2.0, 7.5) |
| indirect |  | 4.7 (0.093, 2.7e+02) |
| network |  | 3.8 (2.1, 7.4) |
| Placebo VS Magnesium |  |  |
| direct | 0.8933 | 4.6 (1.7, 19.) |
| indirect |  | 5.7 (0.49, 66.) |
| network |  | 4.7 (1.9, 15.) |

p < 0.05: significant inconsistency between direct and indirect evidence
