# Supplementary material for: Nebulized pharmacological agents for preventing postoperative sore throat: A systematic review and network meta-analysis
Source: PLoS One. 2020 Aug 10;15(8):e0237174. doi: 10.1371/journal.pone.0237174 (PMC7416917; doi:10.1371/journal.pone.0237174)
Supplement: S1 Appendix — (PDF) [file pone.0237174.s005.pdf]

Systematic review

To edit the record click *Start an update* below. This will create a new version of the record - the existing version will remain unchanged.

1. * Review title.

Give the working title of the review, for example the one used for obtaining funding. Ideally the title should state succinctly the interventions or exposures being reviewed and the associated health or social problems. Where appropriate, the title should use the PI(E)COS structure to contain information on the Participants, Intervention (or Exposure) and Comparison groups, the Outcomes to be measured and Study designs to be included.

Efficacy and acceptability of various nebulized pharmacological agents for prevention of postoperative sore throat: a systematic review and network meta-analysis

2. Original language title.

For reviews in languages other than English, this field should be used to enter the title in the language of the review. This will be displayed together with the English language title.

3. * Anticipated or actual start date.

Give the date when the systematic review commenced, or is expected to commence.

03/03/2020

4. * Anticipated completion date.

Give the date by which the review is expected to be completed.

01/12/2020

5. * Stage of review at time of this submission.

Indicate the stage of progress of the review by ticking the relevant Started and Completed boxes. Additional information may be added in the free text box provided.

Please note: Reviews that have progressed beyond the point of completing data extraction at the time of initial registration are not eligible for inclusion in PROSPERO. Should evidence of incorrect status and/or completion date being supplied at the time of submission come to light, the content of the PROSPERO record will be removed leaving only the title and named contact details and a statement that inaccuracies in the stage of the review date had been identified.

This field should be updated when any amendments are made to a published record and on completion and publication of the review. If this field was pre-populated from the initial screening questions then you are not able to edit it until the record is published.

The review has not yet started: No

Review stage	Started	Completed
Preliminary searches	Yes	Yes
Piloting of the study selection process	Yes	Yes
Formal screening of search results against eligibility criteria	Yes	Yes
Data extraction	Yes	Yes

Review stage	Started	Completed
Risk of bias (quality) assessment	Yes	Yes
Data analysis	Yes	Yes

Provide any other relevant information about the stage of the review here (e.g. Funded proposal, protocol not yet finalised).

6. * Named contact.

The named contact acts as the guarantor for the accuracy of the information presented in the register record.

Jian Yu

Email salutation (e.g. "Dr Smith" or "Joanne") for correspondence:
Dr Yu

7. * Named contact email.

Give the electronic mail address of the named contact.

mryujian@outlook.com

8. Named contact address

PLEASE NOTE this information will be published in the PROSPERO record so please do not enter private information

Give the full postal address for the named contact.

Chongqing Medical University, #1 Yixueyuan Road, Yuzhong District, Chongqing 400016, P. R. China

9. Named contact phone number.

Give the telephone number for the named contact, including international dialling code.

+8618883936873

10. * Organisational affiliation of the review.

Full title of the organisational affiliations for this review and website address if available. This field may be completed as 'None' if the review is not affiliated to any organisation.

Chongqing Medical University

Organisation web address:
https://www.cqmu.edu.cn/

11. * Review team members and their organisational affiliations.

Give the personal details and the organisational affiliations of each member of the review team. Affiliation refers to groups or organisations to which review team members belong. **NOTE: email and country are now mandatory fields for each person.**

Dr Jian Yu. The First Affiliated Hospital of Chongqing Medical University, Chongqing, China
Dr Feng Lv. The First Affiliated Hospital of Chongqing Medical University, Chongqing, China
Dr Li Ren. The First Affiliated Hospital of Chongqing Medical University, Chongqing, China
Dr Yuxi Zhang. The First Affiliated Hospital of Chongqing Medical University, Chongqing, China
Professor Su Min. The First Affiliated Hospital of Chongqing Medical University, Chongqing, China

12. * Funding sources/sponsors.

Give details of the individuals, organizations, groups or other legal entities who take responsibility for initiating, managing, sponsoring and/or financing the review. Include any unique identification numbers assigned to the review by the individuals or bodies listed.

This work was supported by the National Natural Science Foundation of China

Grant number(s)
Grant No. 81873798

13. * Conflicts of interest.

List any conditions that could lead to actual or perceived undue influence on judgements concerning the main topic investigated in the review.

None

14. Collaborators.

Give the name and affiliation of any individuals or organisations who are working on the review but who are not listed as review team members. **NOTE: email and country are now mandatory fields for each person.**

15. * Review question.

State the question(s) to be addressed by the review, clearly and precisely. Review questions may be specific or broad. It may be appropriate to break very broad questions down into a series of related more specific questions. Questions may be framed or refined using PI(E)COS where relevant.

The purpose of our study is to compare the efficacy and acceptability of multiple nebulized drugs (such as glucocorticoids, ketamine, lidocaine, etc.) in preventing postoperative sore throat in patients undergoing endotracheal intubations.

16. * Searches.

State the sources that will be searched. Give the search dates, and any restrictions (e.g. language or publication period). Do NOT enter the full search strategy (it may be provided as a link or attachment.)

We will search the following electronic bibliographic databases: PubMed, EMBASE, The Cochrane Library, and Google Scholar, There will be no language restrictions. We will search the databases from their inceptions to the present. The searches will be re-run just before the final analyses and further studies retrieved for inclusion.

17. URL to search strategy.

Give a link to a published pdf/word document detailing either the search strategy or an example of a search strategy for a specific database if available (including the keywords that will be used in the search strategies), or upload your search strategy.

Do NOT provide links to your search results.

https://www.crd.york.ac.uk/PROSPEROFILES/171703_STRATEGY_20200301.pdf

Do not make this file publicly available until the review is complete

18. * Condition or domain being studied.

Give a short description of the disease, condition or healthcare domain being studied. This could include health and wellbeing outcomes.

Postoperative sore throat, a common complaint of postoperative period after tracheal intubation is being considered as a minor complication, but is a valid reason of dissatisfaction and morbidity among patients.

19. * Participants/population.

Give summary criteria for the participants or populations being studied by the review. The preferred format includes details of both inclusion and exclusion criteria.

The eligible participants are surgical patients who received general anesthesia and endotracheal intubation.

20. * Intervention(s), exposure(s).

Give full and clear descriptions or definitions of the nature of the interventions or the exposures to be reviewed.

Nebulized agents are taken by using an inhaler before intubation or after extubation. Common nebulized agents include corticosteroids, ketamine, lidocaine, magnesium, etc.

21. * Comparator(s)/control.

Where relevant, give details of the alternatives against which the main subject/topic of the review will be compared (e.g. another intervention or a non-exposed control group). The preferred format includes details of both inclusion and exclusion criteria.

Placebo, different kinds of nebulized agents, or no treatment.

22. * Types of study to be included.

Give details of the types of study (study designs) eligible for inclusion in the review. If there are no restrictions on the types of study design eligible for inclusion, or certain study types are excluded, this should be stated. The preferred format includes details of both inclusion and exclusion criteria.

Only randomized controlled study is included.

23. Context.

Give summary details of the setting and other relevant characteristics which help define the inclusion or exclusion criteria.

24. * Main outcome(s).

Give the pre-specified main (most important) outcomes of the review, including details of how the outcome is defined and measured and when these measurement are made, if these are part of the review inclusion criteria.

(1) Incidence and severity of postoperative sore throat after the operation.
(2) adverse effects due to agents.

* Measures of effect
0-24 hours after surgery

25. * Additional outcome(s).

List the pre-specified additional outcomes of the review, with a similar level of detail to that required for main outcomes. Where there are no additional outcomes please state ‘None’ or ‘Not applicable’ as appropriate to the review

1) Scoring system for mucositis in laryngopharynx.
2) Incidence and severity of postoperative cough.
3) Incidence and severity of postoperative hoarseness.

* Measures of effect
0-24 hours after surgery

26. * Data extraction (selection and coding).

Describe how studies will be selected for inclusion. State what data will be extracted or obtained. State how this will be done and recorded.

Data are extracted according to Cochrane handbook for systematic reviews of interventions. All potential articles using the search strategy and those from additional sources will be screened independently by two review authors (Jian Yu AND Feng Lv) to identify whether they meet the inclusion criteria. Full text of potentially

eligible studies are independently assessed for eligibility by two review authors(Li Ren AND Feng Lv). A discussion with a third reviewer(Su Min) will be conducted if any disagreement between them over the eligibility of the studies. A standard form of data extract will be designed and all the information will include: (1) title; (2) authors; (3) name of journal; (4) publication year; (5) study design; (6) registration of clinical trial; (7) competing interests; (8) country; (9) risk of bias; (10) number of patients in study; (11) kinds and doses of drugs compared; (12) sex of patients; (13) age of patients; (14) Incidence and severity of postoperative sore throat after the operation; (15) adverse effects due to agents; (16) Scoring system for mucositis in laryngopharynx; (17) Incidence and severity of postoperative cough; (18) Incidence and severity of postoperative hoarseness. Two review authors(Yuxi Zhang and Jian Yu) will extract data independently, discrepancies will be identified and resolved through discussion. Missing data will be requested from study investigators.

27. * Risk of bias (quality) assessment.

Describe the method of assessing risk of bias or quality assessment. State which characteristics of the studies will be assessed and any formal risk of bias tools that will be used.

The risk of bias were assessed according to Cochrane handbook for systematic reviews of interventions, including selective bias, performance bias, detection bias, attrition bias, reporting bias and other bias. Two review authors (Su Min and Li Ren)will independently assess the risk of bias in included studies as following: Randomization sequence generation: was the randomization sequence generated in proper method? Allocation concealment: was the allocated treatment adequately concealed from study participants and clinicians and other healthcare or research staff at the enrolment stage? Blinding of participants and personnel: were the participants were blind the group and treatment? Blinding of outcome assessment: were the outcome assessors were blind to the group and treatment? Incompleted outcome data: were some results were not reported although it was shown in the methology? Completeness of outcome data: were participant exclusions, attrition and incomplete outcome data adequately addressed in the published report? Selective reporting: is there evidence of selective outcome reporting and might this have affected the study results? Other bias: was the trial apparently free of any other problems that could produce a high risk of bias?

28. * Strategy for data synthesis.

Provide details of the planned synthesis including a rationale for the methods selected. This **must not be generic text** but should be **specific to your review** and describe how the proposed analysis will be applied to your data.

(1) we will conduct a traditional pair-wise comparisons of across comparisons available for each contrast. Due to the expected variation among various nebulized agents, random-effects models will be carried out to compare the efficacy and acceptability of various nebulized agents in each outcome measure. For comparison of continuous outcome and binary outcome, standard mean difference (SMD) and odds ratio (OR) will be calculated both along with 95% credible intervals as the pooled relative effect respectively.
(2) we will conduct a network meta-analysis using a Bayesian approach. Rankograms will be plotted to visualize the relative effectiveness of each intervention for primary outcome. Surface under the cumulative ranking curve (SUCRA) will be used to rank interventions.
(3) A node-splitting model of consistency will be used to check for inconsistencies across the comparisons and the accuracies of the indirect comparisons. In the presence of inconsistency, sensitivity analysis will be performed by removing portions of the network map contributing to inconsistency. Sensitivity analysis was also be conducted by sequentially omitting one study each time.

29. * Analysis of subgroups or subsets.

State any planned investigation of 'subgroups'. Be clear and specific about which type of study or participant will be included in each group or covariate investigated. State the planned analytic approach.

Subgroup analysis shall be conducted in case of high heterogeneity or inconsistency. If enough data are available, we will control for effect modifiers using meta-regression.

30. * Type and method of review.

Select the type of review and the review method from the lists below. Select the health area(s) of interest for your review.

Type of review

Cost effectiveness	No
Diagnostic	No

Epidemiologic	No
Individual patient data (IPD) meta-analysis	No
Intervention	No
Meta-analysis	No
Methodology	No
Narrative synthesis	No
Network meta-analysis	Yes
Pre-clinical	No
Prevention	No
Prognostic	No
Prospective meta-analysis (PMA)	No
Review of reviews	No
Service delivery	No
Synthesis of qualitative studies	No
Systematic review	Yes
Other	No

Health area of the review

Alcohol/substance misuse/abuse	No
Blood and immune system	No
Cancer	No
Cardiovascular	No
Care of the elderly	No
Child health	No
Complementary therapies	No
COVID-19	No
Crime and justice	No
Dental	No
Digestive system	No
Ear, nose and throat	No

Education	No
Endocrine and metabolic disorders	No
Eye disorders	No
General interest	No
Genetics	No
Health inequalities/health equity	No
Infections and infestations	No
International development	No
Mental health and behavioural conditions	No
Musculoskeletal	No
Neurological	No
Nursing	No
Obstetrics and gynaecology	No
Oral health	No
Palliative care	No
Perioperative care	Yes
Physiotherapy	No
Pregnancy and childbirth	No
Public health (including social determinants of health)	No
Rehabilitation	No
Respiratory disorders	No
Service delivery	No
Skin disorders	No
Social care	No
Surgery	No
Tropical Medicine	No
Urological	No
Wounds, injuries and accidents	No
Violence and abuse	No

31. Language.

Select each language individually to add it to the list below, use the bin icon to remove any added in error.

English

There is not an English language summary

32. * Country.

Select the country in which the review is being carried out from the drop down list. For multi-national collaborations select all the countries involved.

China

33. Other registration details.

Give the name of any organisation where the systematic review title or protocol is registered (such as with The Campbell Collaboration, or The Joanna Briggs Institute) together with any unique identification number assigned. (N.B. Registration details for Cochrane protocols will be automatically entered). If extracted data will be stored and made available through a repository such as the Systematic Review Data Repository (SRDR), details and a link should be included here. If none, leave blank.

34. Reference and/or URL for published protocol.

Give the citation and link for the published protocol, if there is one

No I do not make this file publicly available until the review is complete

35. Dissemination plans.

Give brief details of plans for communicating essential messages from the review to the appropriate audiences.

Do you intend to publish the review on completion?

Yes

36. Keywords.

Give words or phrases that best describe the review. Separate keywords with a semicolon or new line. Keywords will help users find the review in the Register (the words do not appear in the public record but are included in searches). Be as specific and precise as possible. Avoid acronyms and abbreviations unless these are in wide use.

systematic review; network meta-analysis; nebulized; postoperative sore throat; Endotracheal Intubation

37. Details of any existing review of the same topic by the same authors.

Give details of earlier versions of the systematic review if an update of an existing review is being registered, including full bibliographic reference if possible.

38. * Current review status.

Review status should be updated when the review is completed and when it is published. For newregistrations the review must be Ongoing.

Review_Ongoing

39. Any additional information.

Provide any other information the review team feel is relevant to the registration of the review.

40. Details of final report/publication(s) or preprints if available.

This field should be left empty until details of the completed review are available OR you have a link to a preprint.
