# Supplementary material for: Nebulized pharmacological agents for preventing postoperative sore throat: A systematic review and network meta-analysis
Source: PLoS One. 2020 Aug 10;15(8):e0237174. doi: 10.1371/journal.pone.0237174 (PMC7416917; doi:10.1371/journal.pone.0237174)
Supplement: S2 Appendix — (DOCX) [file pone.0237174.s006.docx]

**S2 Appendix. Search strategy**

#1 ("Pharyngitis"[Mesh]) OR ((((Pharyngitides[Title/Abstract]) OR Sore Throat[Title/Abstract]) OR Sore Throats[Title/Abstract]) OR Throat, Sore[Title/Abstract])

#2 (sore* OR inflamm* OR infect*) and throat

#3Postoperative sore throat

#4 ("Intubation, Intratracheal"[Mesh]) OR (((((((intratracheal Intubation[Title/Abstract]) OR Intratracheal Intubations[Title/Abstract]) OR Intubations, Intratracheal[Title/Abstract]) OR Intubation, Endotracheal[Title/Abstract]) OR Endotracheal Intubation[Title/Abstract]) OR Endotracheal Intubations[Title/Abstract]) OR Intubations, Endotracheal[Title/Abstract])

#5 #1 OR #2 OR #3 OR #4

#6 inhal*

#7 Aerosoli*

#8 Nebuliz*

#9 #6 OR #7 OR #8

#10 #5 AND #9

#11 randomized controlled trial[Publication Type] OR randomized[Title/Abstract] OR placebo[Title/Abstract]

#12 #10 AND #11
