# Supplementary figures and images for: Nebulized pharmacological agents for preventing postoperative sore throat: A systematic review and network meta-analysis
Source: PLoS One. 2020 Aug 10;15(8):e0237174. doi: 10.1371/journal.pone.0237174 (PMC7416917; doi:10.1371/journal.pone.0237174)

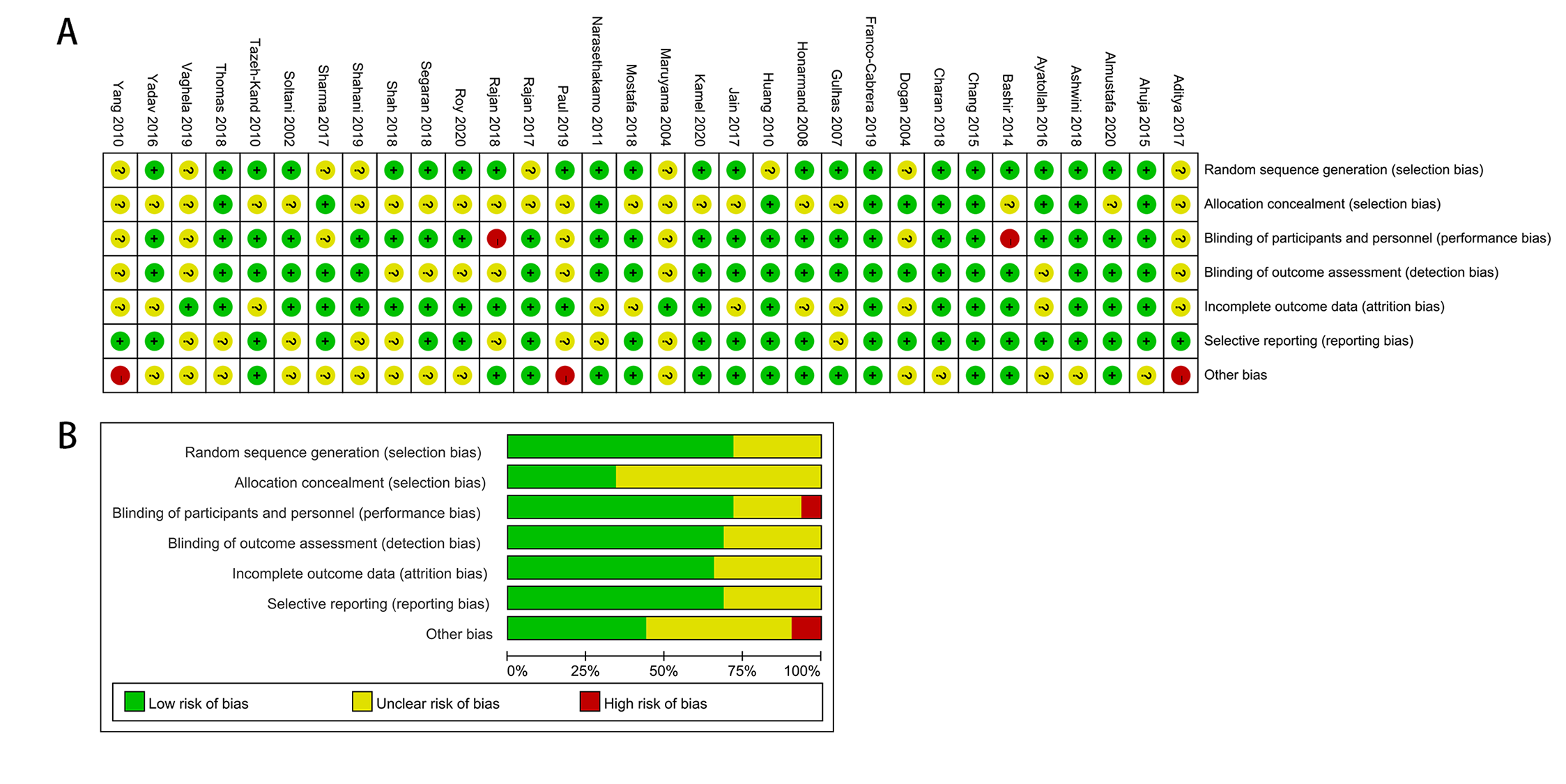

Supplement: S1 Fig — Risk of bias for included studies,(A) Risk of bias summary, (B) Risk of bias graph, green = low risk of bias, yellow = unclear risk of bias, red = high risk of bias. (TIF) [file pone.0237174.s007.tif]
